# Reviewed article: Feitosa YS, Cabral BVB, Lima GS, Gomes EB, Sousa GJB, Moreira TMM, Pereira MLD. Psychosocial risks and work ability among health workers throughout the COVID-19 pandemic: a longitudinal follow-up study, Brazil, 2020-2023. Epidemiol Serv Saude. 2026:35;e20240798

**DOI:** 10.1590/S2237-96222026v35e20240798.a

**Published:** 2026-03-09

**Authors:** Ariane Dini

**Affiliations:** 1 Universidade Estadual de Campinas, Campinas, SP, Brazil

## Peer review A

## Questionnaire

Does the manuscript contain new and significant information to justify publication? Yes

Does the Abstract (Summary) clearly and accurately describe the content of the article? Yes

Is the problem significant and concisely stated? Yes

Are the methods described comprehensively? Yes

Are the interpretations and conclusions justified by the results? Yes

Is adequate reference made to other work in the field? Yes

## Manuscript Structure

Length of article is: Adequate

Number of tables is: Adequate

Number of figures is: Adequate

Please state any conflict(s) of interest that you have in relation to the review of this paper. None

Interest: Excellent

Quality: Good

Originality: Excellent

Overall: Good

Recommendation: Minor Revision

## Comments

Estudo longitudinal prospectivo de 36 meses com objetivo de avaliar riscos psicossociais e a capacidade para o trabalho em profissionais de saúde por 36 meses após o início da pandemia de COVID-19, comparando com profissionais que não são da área de saúde. 

Resumo: Substituir o termo profissionais não saúde por profissionais não relacionados à assistência em saúde (resumo e no decorrer do manuscrito)

Introdução e justificativa: Adequadas, justificam o estudo.

Método:

Rigorosidade metódica adequada com utilização de instrumentos de medida validados na literatura.

Resultados:

Não foi identificado diferença no que se refere à capacidade para o trabalho, no entanto, os riscos psicossociais foram superiores em profissionais da saúde, indicando necessidade de intervenção no ambiente do trabalhador da saúde.

Discussão: Carece de indicação de limitações relacionadas à forma de recrutamento de participantes, diferença de tamanho amostral entre profissionais de saúde e grupo de profissionais de fora da saúde. Embora tenha sido citado de forma descritiva a maior proporção de gênero feminino entre profissionais de saúde, esta variável não foi discutida, nem mesmo quanto a um dos achados sobre atenção sexual indesejada.

Conclusão: Responde aos objetivos.

